# A validated survey to measure Chinese hospital management practices

**DOI:** 10.1016/j.mex.2023.102066

**Published:** 2023-02-05

**Authors:** Hanqing Zhao, Mengxiao Wang, Yujie Cui, Gordon G. Liu

**Affiliations:** a Institute of Health Policy and Hospital Management, Sichuan Provincial People's Hospital, Chengdu, China; b School of Public Administration, Southwestern University of Finance and Economics, Chengdu, China; c China Hospital Development Institute, Shanghai Jiao Tong University, Shanghai, China; d Peking University Institute for Global Health and Development, Beijing, China; e China Center for Health Economic Research, Peking University, Beijing, China

**Keywords:** World management survey, Hospital management, Questionnaire, Measurement, China hospital management survey, Chinese Hospital Management Survey

## Abstract

Surveys are widely used to assess hospital management with the aim of understanding differences in management practices. However, survey measures with prior notice can make hospitals change their routine practices and are unable to reflect the actual hospital management level. The World Management Survey (WMS) methodology has been developed to ameliorate these issues. It uses a double-blinded method and open-ended question design. The Chinese Hospital Management Survey (CHMS) project is the first to adapt the WMS methodology in China, which it uses to measure hospital management level in 510 hospitals. This paper provides an instrument to better measure actual management practices, which makes it possible to compare the management level of hospitals in China and other countries.

## Highlights

• The CHMS followed the WMS methodology, including the scoring of criteria, processes and calibration, to make the measurements comparable with the management scores of hospitals in other countries and across industries using the WMS method.
• The hospital management level generated by the CHMS can distinguish management quality within hospitals in China.
• The quantitative method of hospital management can lay an empirical foundation for future management and economic analysis.

## Graphical abstract

A graphical abstract is mandatory. The graphical abstract should summarize the contents of your article in a concise, pictorial form. Authors must provide images that clearly represent the work described in the article. Graphical abstracts should be submitted as a separate file. Image size: ***please provide an image with a minimum of531 × 1328 pixels (h × w) or proportionally more***. The image should be readable at a size of 5 × 13 cm using a regular screen resolution of 96 dpi. Preferred file types: TIFF, EPS, PDF or MS Office files.

**Figure:**
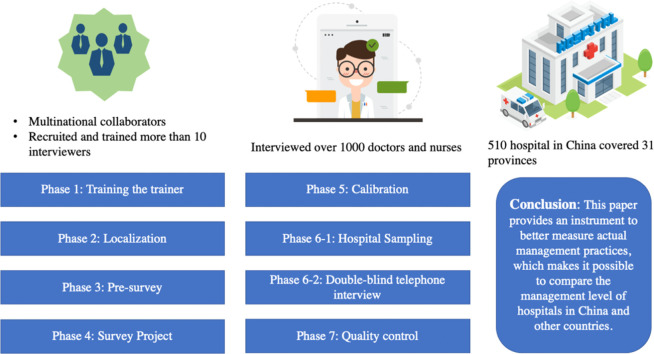

Specifications table

**Table:** 

Subject area:	Economics and Finance

More specific subject area:	Hospital management, management survey
Name of your method:	Chinese Hospital Management Survey
Name and reference of original method:	World Management Survey (1) Measuring and explaining management practices across firms and countries [7] (2) The impact of competition on management quality: evidence from public hospitals [8] (3) Management practices and the quality of care in cardiac units [9]
Resource availability:	The World Management Survey instrument is publicly available at the following web address: https://worldmanagementsurvey.org/

## Method overview

Hospital management is closely related to clinical performance and patient satisfaction [1,2]. As the country faces fast-growing demand and health care expenditures, the establishment of a modern hospital management system to improve the hospital management practices and operational efficiency of Chinese hospitals has been a policy target at the national level [3]. Although the Joint Commission International, German Kooperation fuer Transparenz und Qualität im Gesundheitswesen and Japanese Hospital Function Evaluation Systems, etc., contain many aspects, most of these aspects focus on the evaluation of structure and results. The indicators related to the individual performance of hospital departments occupy a large portion of these systems, while there are almost no effective indicators for the evaluation of hospital management processes; thus, these tools are mostly used for medical quality assessment instead of for evaluating hospital management practices, and they are especially not suitable for quantitative assessment or measurement among hospitals [4]. The hospital accreditation system has been longstanding in China; thus, hospitals are well informed about the standards, procedures and methods and may change their routine practices accordingly [5,6]. To measure the actual management level of hospitals in China, the World Management Survey instrument has been introduced and adapted to Chinese circumstances.

The World Management Survey (WMS), a new quantitative survey methodology proposed by Stanford University economist Nicholas Bloom and LSE economist John Van Reenen, applies a scientific double-blind interview method to comprehensively measure and understand the management level of organizations, and the measurement results are comparable across countries and industries. The WMS is the first cross-country, cross-industry dataset built to measure the quality of management practices in establishments. At its inception, the WMS was only meant for manufacturing industries. However, the survey instrument has now been expanded into the retail, health care, and education industries. Empirical research shows that better management is associated with higher operational efficiency and better quality of care in health care facilities [8,9]. Further experimental research shows that improving management practices could be an effective means of improving the performance of manufacturing firms [10,11].

The Chinese Hospital Management Survey (CHMS) project was initiated through collaboration among Peking University, Tsinghua University, the Chinese Hospital Association, and the Cardiovascular Branch of the Chinese Medical Association. It is the first to introduce and apply the WMS methodology to measure hospital management in China. By following the WMS methodology, we can measure the management level of Chinese hospitals in their natural state, and our results are comparable with other measurements that use the WMS. Additionally, we also add questions related to the characteristics of the health care system and health care reform in China. The CHMS is the first to use a double-blind method to systematically measure the management level of Chinese hospitals and establish an empirical basis for exploring hospital management models in the future.

**Fig. 1:**
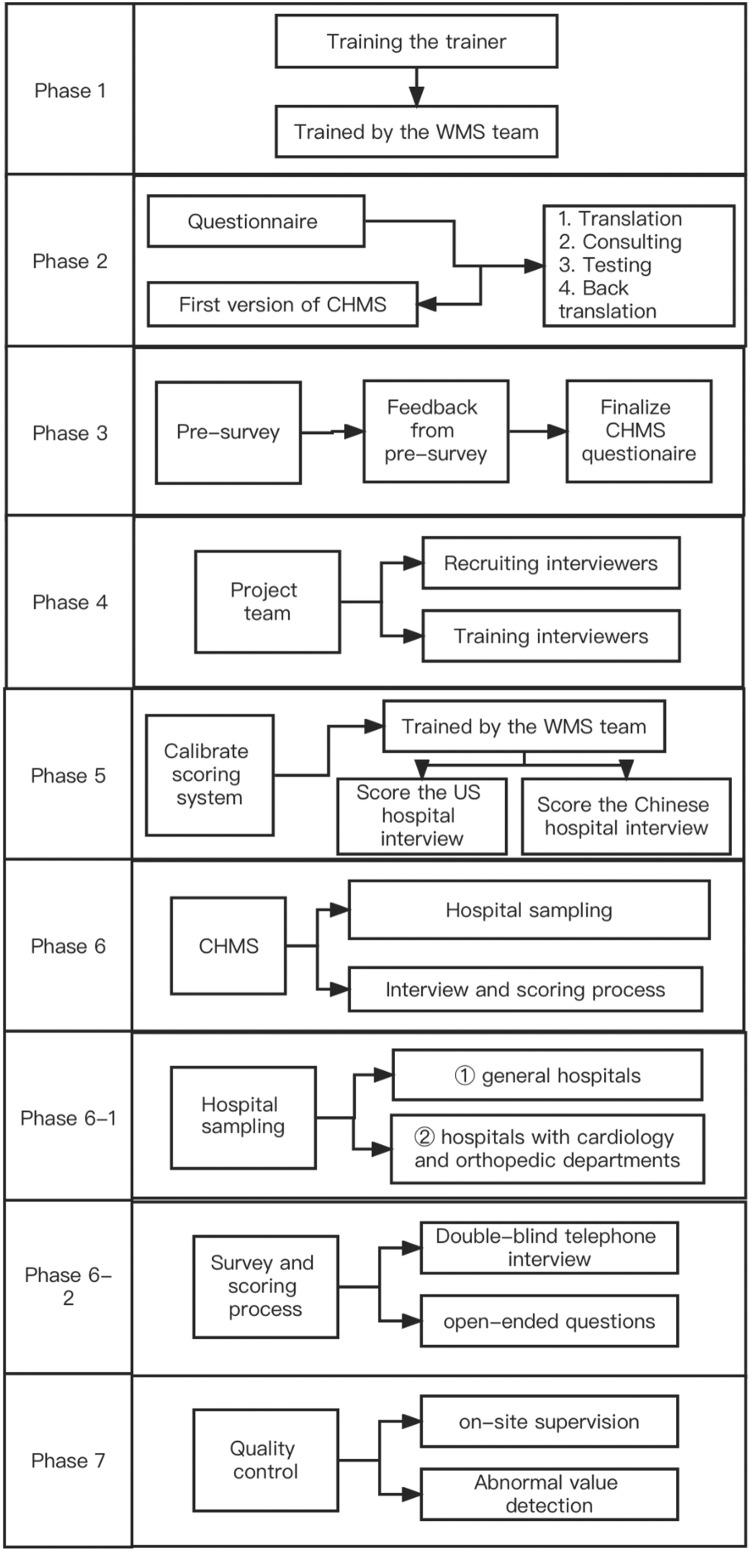
Seven phases of the Chinese Hospital Management Survey.

We structure this technical paper following the major steps of WMS method adaptation. There are seven phases, and each phase contains different steps (see Fig. 1 for details). Phases 1–5 focus on planning the entire project; we do not consider these phases to be uninteresting clichés that should be streamlined. We believe that a thorough approach to preparation will assist international researchers in not becoming overwhelmed when performing projects that utilize new methodologies. Phase 6 focuses on formally carrying out the project, which takes up a significant amount of time for academics. Phase 7 consists of quality control procedures that assist researchers in obtaining accurate data. Following this reproducible pathway of phases assists the research community in adapting the WMS technique to different cultures.

## Procedure

### Training the trainer (Phase one)

After an intensive reading of research papers about the WMS methodology and analysis, we contacted Prof. Nick Bloom's WMS team to start the project training process. The project training process aimed to train the project members with regard to the WMS methodology and project operation. The WMS methodology includes a survey instrument and WMS scoring system. Project operation includes contacting hospitals and interviewees, hiring and training interviewers, and engaging in double-blind interviewing skills. The CHMS project team consists of full-time researchers, postdocs, and some exchange scholars from other universities.

The Chinese Hospital Management Survey methodology is based on WMS articles by Prof. Nick Bloom from Stanford University and his research team from the London School of Economics and Political Science [7], [8], [9]. The survey instrument is based on the WMS guidelines for medical institutions (http://worldmanagementsurvey.org/survey-data/methodology).

### Questionnaire (Phase two)

After the project training process, the next phase was questionnaire adaptation. The steps of this phase were as follows:

• Translation. The first step was to translate the questionnaire directly from English to Chinese. Here, the emphasis should be solely on grammar checks from the original language to the chosen language, such as sentence components, and the meaning of professional jargon should be left to experts to discuss.
• Consultation. We consulted with Chinese hospital management experts, hospital management researchers and clinicians or managers to advise on the hospital management and medical-related terms used in the questionnaire. We summarized the comments from experts and discussed possible modifications.
• Testing. We tested the Chinese translation among team members. We found that some of the translated questions were confusing to the Chinese respondents; thus, we made adjustments to the questioning style to align with the Chinese linguistic and cultural environment.
• Back-translation. The collated Chinese questionnaire was translated into English and sent back to the WMS team to ensure the consistency of meaning in the survey instrument. When there were disagreements during the back translation, modifications were made unless the meaning was entirely different or there was ambiguity. We aimed to make minor modifications in this stage rather than large ones.

Through this process, we localized the WMS survey instrument and obtained the first version of the China Hospital Management Survey (CHMS) questionnaire. However, the feasibility of the instrument needed to be tested in the real world before formal interviews were conducted.

### Presurvey (Phase three)

In the CHMS project, we used double-blind telephone interviews to obtain the true level of management practices in the natural state of the hospital and to reduce interviewer-respondent bias. First, the interviewer did not know relevant information about the interviewee (e.g., name of the interviewed hospital, rank, performance, etc.) prior to the interview, which aimed to eliminate bias in scoring due to the interviewer's preconceived notions. Second, the interviewees did not know the interviewer's appearance, background, questionnaire content or rating. Our interviews focused the interviewees' daily work; thus, we did not need the interviewees to prepare in advance, which reduced the psychological pressure of the interviewees and enabled the interviewees to reflect on their management practices more realistically.

Through convenience sampling, we conducted telephone interviews in 20 general hospitals from 12 provinces (1 in Heilongjiang, 1 in Jilin, 1 in Liaoning, 1 in Shandong, 1 in Jiangsu, 1 in Fujian, 3 in Guangdong, 1 in Hubei, 1 in Shanxi, 1 in Guizhou, 2 in Gansu, and 1 in Qinghai) and 2 municipalities (4 in Beijing and 1 in Chongqing). In this stage, we found that hospitals were willing to participate in our project and could understand our questions on management practices, which made us confident regarding the feasibility of the CHMS method.

Based on the feedback received from the presurvey, we further optimized the survey process and made targeted adjustments to contextualize the questionnaire. There were several modifications made. First, considering the fact that Chinese hospitals have different management systems from those used in foreign countries, we removed the questions related to unions and hospital directors' salaries. This was because most public hospital directors are appointed by relevant local government health departments and are paid mainly according to their official ranking and hospital performance, not market prices; thus, their salaries cannot be directly compared with directors' salaries obtained from WMS interviews conducted at U.S. hospitals. Second, based on discussions with Chinese hospital managers and research scholars, we added open-ended questions with Chinese characteristics, such as the reform of separation of management and administration, the degree of autonomy in decision-making, the method of health insurance payment, the method of bonus distribution, and the personnel system arrangement. These questions were in addition to the management survey and featured the recent reform of public hospitals to further reflect some of the hot spots and key issues of hospital management reform in China.

After finalizing the abovementioned modification work, we completed the localization and adjustment of the WMS instrument. The survey instrument contains two main parts: the hospital management level survey part and the hospital information collection part (including basic information about hospitals and departments, as well as questions related to hospital management reform). The CHMS instrument includes 20 management practices covering four dimensions of hospital management, namely, operational management, performance monitoring, target management, and talent management (see Appendices A and B). Based on the interviewees' responses and the CHMS scoring criteria, the interviewers scored each management practice ranging from 1 to 5 (1 for the worst and 5 for the best).

### Training interviewers (Phase four)

Since this survey project required a high overall ability of interviewers, the CHMS project team recruited outstanding students with backgrounds in economics, sociology, and management from top universities to serve as interviewers. The nine selected interviewers received a week-long intensive training by the CHMS hospital management project team and hospital management experts to better comprehend the connotations of the hospital management survey instrument questionnaire by understanding the daily operations in hospitals, which would in turn help in the interviewing and scoring process. During the training, the project team was responsible for theoretical training on survey methodology, telephone interview techniques, key questions, scoring criteria, etc.

### Scoring calibration (Phase five)

The CHMS project team and interviewers worked with the WMS team to calibrate the CHMS scoring system to ensure that the CHMS survey results were comparable with the WMS scores. The WMS team shared their global experience in conducting hospital management surveys. To address the scoring consistency issues due to the large cultural and language differences between English and Chinese, the following two steps were taken:

• To understand how the WMS team conducted the interviews and scored the hospital management practices, both the CHMS project team and interviewers listened to the full telephone interviews and scoring discussion process conducted by the WMS team for U.S. hospitals online. At the same time, CHMS interviewers scored the US hospitals' interviews. Through discussions with the WMS team, CHMS members learned specific scoring points and formed a consensus with the WMS team, thus ensuring that the scoring criteria were the same.
• To test whether the CHMS interview scoring was consistent with the WMS interview, the CHMS team translated the interview transcripts from Chinese hospitals into English and transmitted them to the WMS team for parallel scoring. Then, we compared and analyzed both scores and further unified the scoring criteria.

### Formal interview (Phase six)

After the adaptation of the survey questionnaire and the calibration of the scoring system, we started our formal interviews. These interviews were mainly composed of sampling and telephone interviews.

### Sampling

Our target hospitals were general hospitals in mainland China with cardiology and orthopedic departments (for some hospitals without cardiology or orthopedic departments, we selected their major internal medicine or major surgery departments). We obtained the contact information of the hospitals through collaborations with Tsinghua University, the Chinese Hospital Association and many other organizations. Our sampling frame was mainly based on the hospital list obtained from the Chinese Hospital Association, which contains the name, address, grade and phone number of most hospitals in mainland China. We also contacted hospital managers who attended training courses organized by partner organizations to determine whether they would be interested participating in our project.

**Fig. 2:**
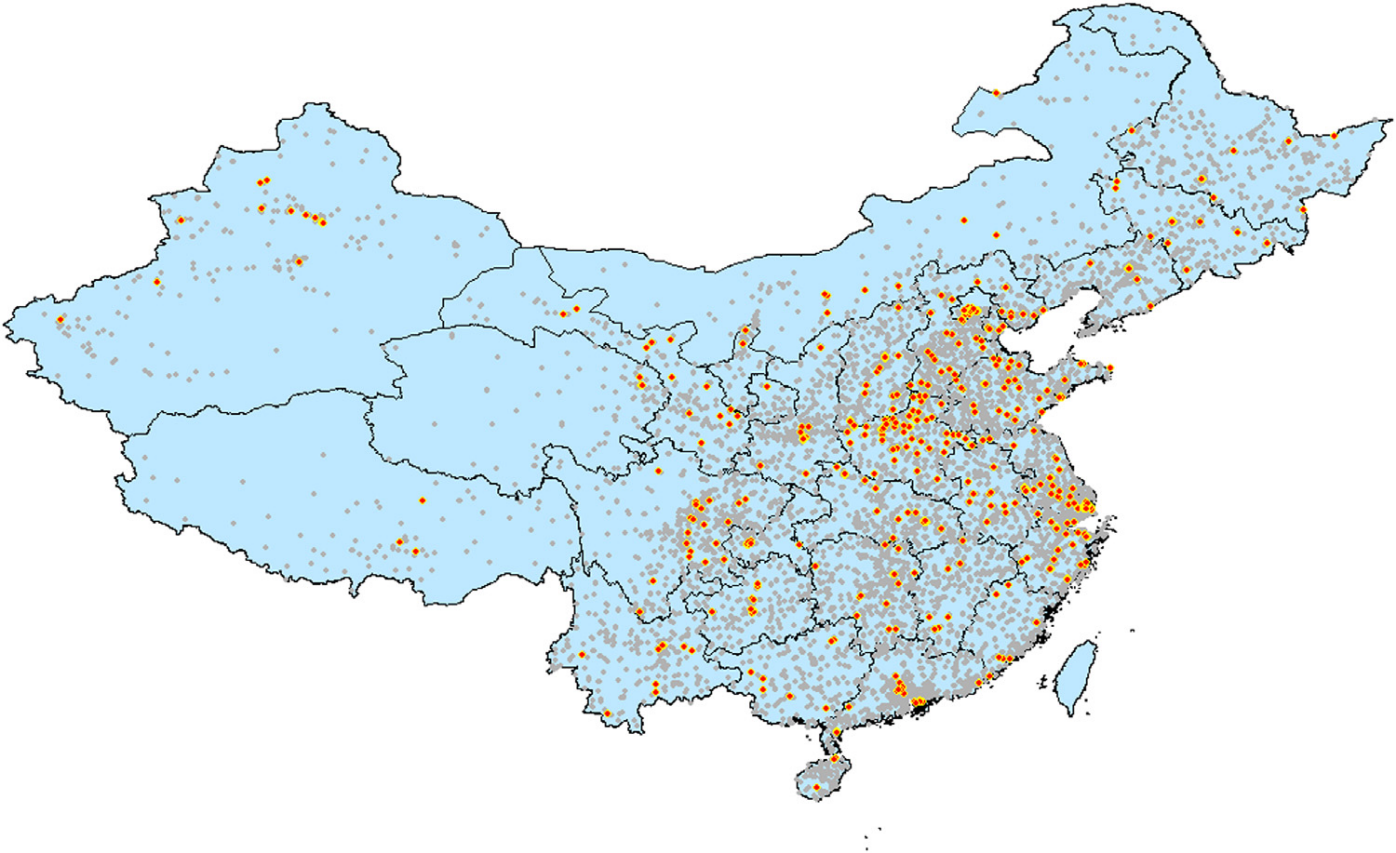
Distribution of Hospitals in the Chinese Hospital Management Survey.

Note: Gray points indicate the hospitals listed in the Chinese Hospital Association; red points indicate the hospitals that completed the survey.

A convenience sampling method was used, and the sample hospitals covered 31 provinces, cities and autonomous regions in mainland China. We contacted 2783 target hospitals. The final sample of successful interviews was 264 tertiary hospitals, 227 secondary hospitals, and 19 primary hospitals, for a total of 510 hospitals and a response rate of approximately 19% (see Fig. 2 for the distribution of hospitals in the CHMS).

To understand the real situation of hospital management, we chose middle-level hospital managers as interviewees, including the heads of clinical department management, some hospital function managers, directors of cardiology and orthopedic departments, head nurses, and the heads of the medical departments and nursing departments of some hospitals. These interviewees could understand the overall operation and management of the hospital at a macro level and were familiar with the microlevel daily management affairs of the hospital, as well as clinical departments. The number of interviewees from each hospital ranged from one to four.

#### Interview and scoring process

To reduce the rejection rate, the interviewing team made prior telephone contact with all hospitals in the sample to declare the purpose of the survey, to gain trust and permission for interviewing and to ask each hospital to recommend qualified interviewees. Then, the interviewer team scheduled a convenient time to contact each recommended interviewee.

When we contacted the interviewees, we were straightforward in saying that the interviews would only be used to study hospital management, with the aim that the interviewees would not change the content and tone of their answers for various reasons.

### Example text of the preannouncement

**Table:** 

Hello, I am an interviewer for the Global Hospital Management Survey at the China Center for Health Economics Research, Peking University. We have contacted your hospital, and they have recommended you for this interview. Our interview will last 30–40 min and will not be recorded. We will take notes for academic research purposes only, so please feel free to speak up. Do you have any questions before the interview officially begins? Then, the interview begins.

To avoid leading respondents to exaggerate, interviewers used three to five open-ended questions to collect information for each management practice and encouraged respondents to give specific examples. The scores for each management practice were arithmetically averaged to create a hospital management level score. Hospital-related information was collected mainly through structured questions (basic hospital and department information) and a small number of open-ended questions (health care reform-related questions).

After investigating the 20 management practices, we also collected objective information in the category of hospital organization through the use of closed-ended questions. This objective information was not used to affect the hospital management level score.

The interviews lasted for approximately 40 min on average, and each interview was attended by at least two interviewers. One interviewer was responsible for asking questions and was allowed to flexibly change the questioning style and orders according to the respondent's answers. Meanwhile, the other interviewer acted as a recorder and was responsible for listening and taking rigorous records of the interview process, including the interview time, interview length, the respondent's knowledge of management practices, and his or her willingness to provide information. The two interviewers independently scored each management practice. Afterward, the interviewers discussed the management practices with inconsistent scores and developed a unified score for the interviewee's management score. Finally, the management scores of all interviewees in the hospital were arithmetically averaged to form the hospital management score.

### Quality control (Phase seven)

The CMHS project team, as quality control supervisors of the interview process, randomly selected approximately 10% of the interview cases for on-site supervision. In addition to on-site interview supervision, the project team also performed quality control work. When scoring or other anomalies (such as data typos and missing values) were found, we traced the issues back to the original interview transcripts and original data sheets to analyze the mistakes. Adjustments were made accordingly after discussions were held with the interviewers and recorders.

## Reliability and validity analysis

The formal interviews began in September 2014 with four rounds of surveys, and they ended in January 2017. After we had collected enough interview samples, we conducted reliability and validity analyses using a sample of 810 interviewees from 424 hospitals. Details about the testing process have been published elsewhere [12].

We examined the internal reliability by using SPSS 19 statistical software, specifically the split-half reliability test and Cronbach's alpha test. In the split-half reliability test, the CHMS earned approximately 0.9. In Cronbach's alpha test, the CHMS yielded a coefficient of 0.920. The results indicate that the CHMS survey method has good internal reliability.

As the WMS was mainly developed by researchers based on the situation in the United States, Europe, and some third-world countries, we also tested the CHMS regarding its validity in the Chinese hospital setting. We used confirmatory factor analysis (CFA) to analyze the structural validity. The CHMS yielded a goodness-of-fit index (GFI) of 0.956 and a root mean square residual (RMR) of 0.015, indicating good structural validity.

## Conclusions and future research

In this paper, we illustrated a comprehensive process for preparing, localizing, and conducting the WMS methodology in Chinese hospitals. We provided a procedure to adapt the WMS in a different culture while ensuring the consistency of measurements; furthermore, this approach is replicable in other countries.

We encourage all social science researchers to use the WMS quantitative management technique to conduct research using previously difficult-to-measure data in the field of management. From a methodological perspective, we hope to support the research community by providing our technical recommendations for WMS research on hospital management levels. At the same time, we advocate further innovation in the development of quantitative management techniques.

## Related research article

Mengxiao Wang, Thomas Butt, Gordon G Liu, Nicholas Bloom, Hanqing Zhao, Yujie Cui, Maorui Yang, Tingfang Liu. Measuring and explaining management practices in Chinese hospitals, under revision, *Social Science and Medicine*

## Funding

This work was supported by the National Science Foundation of China (grant number: 71773002), 10.13039/100001547China Medical Board (grant numbers: 12–109, 15–217), and Boston Scientific Funds (grant number: 050196).

## CRediT authorship contribution statement

**Hanqing Zhao:** Visualization, Investigation, Methodology, Writing – original draft, Supervision. **Mengxiao Wang:** Software, Writing – review & editing. **Yujie Cui:** Formal analysis, Data curation, Supervision. **Gordon G. Liu:** Conceptualization, Writing – review & editing.

## Declaration of Competing Interest

The authors declare that they have no known competing financial interests or personal relationships that could have appeared to influence the work reported in this paper.

## Data Availability

Data will be made available on request.

## Appendix Supplementary materials

Image, application 1

## References

[1] Wang M., Liu G.G.E., Bloom N. (2022). Medical disputes and patient satisfaction in China: how does hospital management matter?. Int. J. Health Plann. Manag..

[2] McConnell K.J., Lindrooth R.C., Wholey D.R., Maddox T.M., Bloom N. (2016). Modern management practices and hospital admissions. Health Econ..

[3] General Office of the State Council. Guiding opinions of the general office of the state council on establishing a modern hospital management system. Accessed June 16, 2022. http://www.lawinfochina.com/display.aspx?id=26294&lib=law&EncodingName=big5

[4] Ruya Guo, Zhang Sonia, Lixia Dou., Yifei Zhao, Xing Zhang, Yangfeng Wu (2015). Process evaluation of hospital management practices. Chinese Hospitals.

[5] Liu T. (2011). New Hospital Accreditation without path dependence. China Hospital CEO.

[6] National Health Commission. Tertiary hospital accreditation criteria (2020). Accessed March 19, 2021. http://www.gov.cn/zhengce/zhengceku/2020-12/28/content_5574274.htm

[7] Bloom N., Van Reenen J. (2007). Measuring and explaining management practices across firms and countries. Q. J. Econ..

[8] Bloom N., Propper C., Seiler S., Van Reenen J. (2015). The impact of competition on management quality: evidence from public hospitals. Rev. Econ. Stud..

[9] McConnell K.J., Lindrooth R.C., Wholey D.R., Maddox T.M., Bloom N. (2013). Management practices and the quality of care in cardiac units. JAMA Intern. Med..

[10] Bloom N., Eifert B., Mahajan A., McKenzie D., Roberts J. (2013). Does management matter? Evidence from India. Q. J. Econ..

[11] Bloom N., Mahajan A., McKenzie D., Roberts J. (2020). Do management interventions last? Evidence from India. Am. Econ. J.: Appl. Econ..

[12] Cui Y., Zhao H., Liu G., He Y., Wang M., Yang M. (2018). Study on the establishment of the internationally comparable Chinese hospital management survey system. China Health Policy Res..

