# Supplementary material for: A validated survey to measure Chinese hospital management practices
Source: MethodsX. 2023 Feb 5;10:102066. doi: 10.1016/j.mex.2023.102066 (PMC9975700; doi:10.1016/j.mex.2023.102066)
Supplement: Supplementary file 1 [file mmc1.docx]

Supplementary materials-CHMS questionnaire (English and Chinese version)

**Table A: CHMS questionnaire framework in English**

| **1) Layout of Patient Flow** | | | |
| --- | --- | --- | --- |
| Tests how well the patient pathway is configured at the infrastructure level and whether staff pro-actively improve their own work-place organization | | | |
|  | a) Can you briefly describe the patient journey or flow for a typical episode? | | |
|  | b) How closely located are wards, theatres, diagnostics centers and consumables? Is there any elevator only for patients? | | |
|  | c) How often do you run into problems with the current layout and pathway management? | | |
|  | d) Has the patient flow and the layout of the hospital challenged/changed? Can you think of any examples? | | |
| **Scoring grid:** | **Score 1:** Lay-out of hospital and organization of workplace is not conducive to patient flow (e.g., ward is on different level from theatre or consumables are often not available in the right place at the right time) | **Score 3:** Lay-out of hospital has been thought-through and optimized as far as possible; work place organization is not regularly challenged/ changed (or vice versa) | **Score 5:** Hospital layout has been configured to optimize patient flow; workplace organization is challenged regularly and changed whenever needed |
| **2) Rationale for Introducing Standardization/ Pathway Management** | | | |
| Tests the motivation and impetus behind changes to operations and what change story was communicated | | | |
|  | a) Can you take me through the rationale for making operational improvements to the management of the patient pathway? Can you describe a recent example? | | |
|  | b) How often do you challenge/ streamline the patient pathway? | | |
|  | c) What factors led to the adoption of these practices? Who typically drives these changes? | | |
|  | d) Will the affected staff groups know the rationale behind changes? How is the participation level? Can you describe an example of changes promoted by the staff? | | |
| **Scoring grid:** | **Score 1:** Changes were imposed top down or because other departments were making (similar) changes; rationale was not communicated or understood | **Score 3:** Changes were made because of financial pressure and the need to save money or as a (short-term) measure to achieve government and/ or external targets | **Score 5:** Changes were made to improve overall performance, both clinical and financial, with buy-in from all affected staff groups; the changes were communicated in a coherent ‘change story’ |
| **3) Standardization and Protocols** | | | |
| Tests if there are standardized procedures (e.g., integrated clinical pathways) that are applied and monitored systematically | | | |
|  | a) How standardized are the main clinical processes? Can you think of any examples? | | |
|  | b) How clear are clinical staff members about how specific procedures should be carried out? | | |
|  | c) How are managers able to monitor whether clinical staff are following established protocols? | | |
| **Scoring grid:** | **Score 1:** Little standardization and few protocols exists (e.g., different clinical staff have different approaches to the same treatments) | **Score 3:** Protocols have been created, but are not commonly used because they are too complicated or not monitored adequately (e.g., may be on website or in manual only) | **Score 5:** Protocols are known and used by all clinical staff and regularly followed up on through some form of monitoring or oversight |
| **4) Continuous Improvement** | | | |
| Tests processes for and attitudes towards continuous improvement, and whether learnings are captured and documented | | | |
|  | a) How do problems typically get exposed and fixed? Can you talk me through the process for a recent problem that you faced? | | |
|  | b) When processes do change, what is the main driver of change? | | |
|  | c) Who within the hospital typically gets involved in changing or improving? How do/ can different staff groups get involved in this process? | | |
| **Scoring grid:** | **Score 1:** Process improvements are made only when problems occur, or only involve one staff group | **Score 3:** Improvements are made in irregular meetings involving all staff groups, to improve performance in their area of work (e.g., ward or theatre) | **Score 5:** Exposing problems in a structured way is integral to individuals’ responsibilities and resolution involves all staff groups, along the entire patient pathway; exposing and resolving problems is a part of a regular business process rather than being the result of extraordinary efforts |
| **5) Good use of Human Resources** | | | |
| Tests whether staff are deployed to do what they are best qualified for, but nevertheless help out elsewhere when needed | | | |
|  | a) How do you know which tasks are best suited to different staff? | | |
|  | b) With respect to your staff, what happens when different hospital areas become busier than others? | | |
|  | c) What kind of procedures do you have in place to assist staff flow between areas; for example, is there one central person or center which coordinates this process? | | |
| **Scoring grid:** | **Score 1:** Staff often end up undertaking tasks for which they are not qualified or over-qualified when they could be used elsewhere; staff do not move across units, even when they are generally underutilized | **Score 3:** Senior staff try to use the right staff for the right job, but do not go to great lengths to ensure this; staff may move but often in an uncoordinated manner | **Score 5:** Staff recognize effective human resource deployment as a key issue and will go to some lengths to make it happen; shifting staff from less busy to busy areas is done routinely and in a coordinated manner, based on the documented skills |
| **6) Performance Tracking** | | | |
| Tests whether performance is tracked using meaningful metrics and with appropriate regularity | | | |
|  | a) What kind of performance or quality indicators would you use for performance tracking? Which indicators are relatively important? | | |
|  | b) How frequently are these measured? Is there any department or person responsible for this work? | | |
|  | c) Who gets to see these data? | | |
|  | d) If I were to walk through your hospital wards and surgical rooms, could I tell how you were doing against your performance goals? How frequently are these updated? | | |
| **Scoring grid:** | **Score 1:** Measures tracked do not indicate directly if overall objectives are being met (only government targets are tracked); tracking is an ad-hoc process (certain processes aren’t tracked at all) | **Score 3:** Most important performance or quality indicators are tracked formally; tracking is overseen by senior staff | **Score 5:** Performance or quality indicators are continuously tracked and communicated against most critical measures, both formally and informally, to all staff using a range of visual management tools |
| **7) Performance Review** | | | |
| Tests whether performance is reviewed with appropriate frequency and communicated to staff | | | |
|  | a) How do you review your main performance indicators? | | |
|  | b) How frequently is performance reviewed? Who gets to see the results of this review? | | |
|  | c) What is a typical follow-up plan that results from performance review? | | |
| **Scoring grid:** | **Score 1:** Performance is reviewed infrequently or in an un-meaningful way (e.g., only success or failure is noted) | **Score 3:** Performance is reviewed periodically with both successes and failures identified; results are communicated to senior staff; no clear follow up plan is adopted | **Score 5:** Performance is continually reviewed, based on the indicators tracked; all aspects are followed up on, to ensure continuous improvement; results are communicated to all staff |
| **8) Performance Dialogue** | | | |
| Tests the quality of review conversations | | | |
|  | a) Have you ever participated in the performance review meetings? How are these meetings structured? How is the agenda determined? | | |
|  | b) Who is involved and what information are provided in these meetings? During these meetings do you find that you generally have enough information for review? | | |
|  | c) How useful do you find these meetings? What type of feedback occurs in these meetings? | | |
|  | d) For a given problem, how do you generally identify the root cause? | | |
| **Scoring grid:** | **Score 1:** The right information for a constructive discussion is often not present or the quality is too low; conversations focus overly on data that is not meaningful; a clear agenda is not known and purpose is not explicitly stated; next steps are not clearly defined | **Score 3:** Review conversations are held with the appropriate data present; objectives of meetings are clear to all participating and a clear agenda is present; conversations do not, drive to the root causes of the problems; next steps are not well defined | **Score 5:** Regular review/performance conversations focus on problem solving and addressing root causes; purpose, agenda and follow-up steps are clear to all; meetings are an opportunity for constructive feedback and coaching |
| **9) Consequence Management** | | | |
| Tests whether differing levels of performance (NOT personal but plan/ process based) lead to different consequence | | | |
|  | a) Let’s say you’ve agreed to a follow-up plan at one of your meetings, how do you know the progress of the plan? | | |
|  | b) What would happen if the plan weren’t enacted? | | |
|  | c) How long is it between when a problem is identified to when it is solved? Can you give me a recent example? | | |
|  | d) How do you deal with repeated failures in a specific sub-specialty or cost area? | | |
| **Scoring grid:** | **Score 1:** Failure to achieve agreed objectives does not carry any consequences | **Score 3:** Failure to achieve agreed results is tolerated for a period before action is taken | **Score 5:** A failure to achieve agreed targets drives retraining in identified areas of weakness or moving individuals to where their skills are appropriate |
| **10) Target Balance** | | | |
| Tests whether targets cover a sufficiently broad set of metrics | | | |
|  | a) What types of targets are set for the hospital? Which goals are emphasized more often? | | |
|  | b) Tell me about goals that are not set externally (e.g., by the government, regulators)? | | |
|  | c) What is the relationship between these goals? | | |
| **Scoring grid:** | **Score 1:** Goals focused only on government targets and achieving the budget | **Score 3:** Goals are balanced set of targets (including quality, waiting time, operational efficiency, and financial balance); goals form part of the appraisal for senior staff only or do not extend to all staff groups; real interdependency is not well understood | **Score 5:** Goals are a balanced set of targets covering all four dimensions (see Score 3); interplay of all four dimensions is understood by senior and junior staff (clinicians as well as nurses and managers) |
| **11) Target Inter-Connection** | | | |
| Tests whether targets are tied to hospital objectives and how well they cascade down the organization | | | |
|  | a) What is the motivation behind these goals? | | |
|  | b) How are these goals cascaded down to the different staff groups or to individual staff members? | | |
|  | c) How are your unit targets linked to overall hospital performance and its goals? Can you think of any examples? | | |
| **Scoring grid:** | **Score 1:** Goals do not cascade down the organization | **Score 3:** Goals do cascade, but only to some staff groups (e.g., nurses only) | **Score 5:** Goals increase in specificity as they cascade, ultimately defining individual expectations for all staff groups |
| **12) Time Horizon of Targets** | | | |
| Tests whether hospital has a ‘3 horizons’ approach to planning and targets | | | |
|  | a) What kind of time scale are you looking at with your targets? Which goals receive the most emphasis? | | |
|  | b) What is the relationship between long-term and short-term goals? | | |
|  | c) Could you meet all your short-run goals but miss your long-run goals? | | |
| **Scoring grid:** | **Score 1:** The staff’s main focus is on achieving short-term targets | **Score 3:** There are short and long-term goals for all levels of the organization; goals are set independently and therefore are not necessarily linked to one another | **Score 5:** Long-term goals are translated into specific short-term targets so that short-term targets become a ‘staircase’ to reach long-term goals |
| **13) Target Stretch** | | | |
| Tests whether targets are appropriately difficult to achieve | | | |
|  | a) How tough are your targets? How pushed are you by the targets? On average, how often would you say that you meet your targets? | | |
|  | b) How are your targets benchmarked? | | |
|  | c) Do you feel all specialties, departments or staff groups receive the same degree of difficulty in terms on targets? Do some groups perhaps have easier targets? | | |
| **Scoring grid:** | **Score 1:** Goals are either too easy or impossible to achieve, at least in part because they are set with little clinician involvement (e.g., simply off historical performance) | **Score 3:** In most areas, senior staff push for aggressive goals based on external benchmarks, but with little buy-in from clinical staff; there are a few sacred cows that are not held to the same standard | **Score 5:** Goals are genuinely demanding for all parts of the organization and developed in consultation with senior staff (e.g., to adjust external benchmarks appropriately) |
| **14) Clarity and Comparability of Targets** | | | |
| Tests how easily understandable performance measures are and whether performance is openly communicated | | | |
|  | a) If I asked someone on your staff directly about individual targets, what would he or she tell me? | | |
|  | b) Does anyone complain that the targets are too complex? | | |
|  | c) How do people know how their own performance compares to other people’s performance? Is this published or posted in any way? | | |
| **Scoring grid:** | **Score 1:** Performance measures are complex and not clearly understood, or only relate to government/regulator targets; individual performance is not made public | **Score 3:** Performance measures are well defined and communicated; performance is public at all levels but comparisons are discouraged | **Score 5:** Performance measures are well defined, strongly communicated and reinforced at all reviews; performance and rankings are made public to induce competition |
| **15) Rewarding High Performers** | | | |
| Tests whether good performance is rewarded proportionately | | | |
|  | a) How does your appraisal/ review system work? Can you tell me about your most recent round? | | |
|  | b) How does your staff’s pay relate to the results of this review? How does the bonus system work? | | |
|  | c) Are there non-financial rewards for the best performers across all staff groups? | | |
|  | d) How does your reward system compare to that at other comparable hospitals? | | |
| **Scoring grid:** | **Score 1:** Staff members are rewarded in the same way irrespective of their level of performance | **Score 3:** There is an evaluation system for the awarding of performance related rewards that are non-financial at the individual level; rewards are always or never achieved | **Score 5:** There is an evaluation system which rewards individuals based on performance; the system includes both personal financial and non-financial awards; rewards are awarded as a consequence of well-defined and monitored individual achievements |
| **16) Removing Poor Performers** | | | |
| Tests whether hospital is able to deal with underperformers | | | |
|  | a) If you had a clinician or a nurse who could not do his/her job, what would you do? Could you give me a recent example? | | |
|  | b) How long is under-performance tolerated? | | |
|  | c) How difficult is it to terminate a nurse/ clinician? | | |
|  | d) Do you find staff members who lead a sort of charmed life? Do some individuals always just manage to avoid being fired? | | |
| **Scoring grid:** | **Score 1:** Poor performers are rarely removed from their positions | **Score 3:** Suspected poor performers stay in a position for more than a year before action is taken | **Score 5:** We move poor performers out of the hospital/ department or to less critical roles as soon as a weakness is identified |
| **17) Promoting High Performers** | | | |
| Tests whether promotion is performance based | | | |
|  | a) Can you tell me about your career progression/ promotion system? How frequently is the promotion? | | |
|  | b) How do you identify and develop your star performers? What types of professional development opportunities are provided? | | |
|  | c) How do you make decisions regarding progression/ promotions within the unit/ hospital? | | |
|  | d) Are better performers likely to be promoted faster or are promotions given on the basis of tenure/ seniority? | | |
| **Scoring grid:** | **Score 1:** People are promoted primarily on the basis of tenure (years of service) | **Score 3:** People are promoted upon the basis of performance | **Score 5:** We actively identify, develop and promote our top performers |
| **18) Managing Talent** | | | |
| Tests what emphasis is put on talent management | | | |
|  | a) How do you ensure you have enough staff/ nurses of the right type in the hospital? | | |
|  | b) How do senior managers show that attracting talented individuals and developing their skills is a top priority? | | |
|  | c) Do senior staff members get any rewards for bringing in and keeping talented people in the hospital? | | |
| **Scoring grid:** | **Score 1:** Senior staff do not communicate that attracting, retaining and developing talent throughout the organization is a top priority | **Score 3:** Senior staff believe and communicate that having top talent throughout the organization is key to good performance | **Score 5:** Senior staff are evaluated and held accountable on the strength of the talent pool they actively build |
| **19) Retaining Talent** | | | |
| Tests whether hospital will go out of its way to keep its top talent | | | |
|  | a) If you had a top performing manager, nurse or clinician that wanted to leave, what would the hospital do? | | |
|  | b) Could you give me an example of a star performer being persuaded to stay after wanting to leave? | | |
| **Scoring grid:** | **Score 1:** We do little to try and keep our top talent | **Score 3:** We usually work hard to keep our top talent | **Score 5:** We do whatever it takes to retain our top talent across all staff groups |
| **20) Attracting Talent** | | | |
| Tests the strength of the employee value proposition | | | |
|  | a) What makes it distinctive to work at your hospital, as opposed to other similar hospitals? | | |
|  | b) If I were a top nurse/clinician and you wanted to persuade me to work at your hospital, how would you do this? | | |
|  | c) What do you think people may not like about working at your hospital? | | |
|  | d) How would you rate your well-being at work? From 1 to 5, and 5 indicates the best. | | |
| **Scoring grid:** | **Score 1:** Competing hospitals offer stronger reasons for talented people to join their organizations | **Score 3:** Our value proposition is comparable to those offered by other hospitals | **Score 5:** We provide a unique value proposition to encourage talented individuals to join our hospital before our competition |

*Note: each question has 5 levels with a minimum score of 1 and maximum score of 5. Domain score is calculated by taking an average across the domain questions. Total score is calculated by taking an average across all 20 questions.*

**Table B: CHMS questionnaire framework in Chinese**

| **管理类问题*** | | | | | | | | | | |
| --- | --- | --- | --- | --- | --- | --- | --- | --- | --- | --- |
| 一、医院布局  - 询问门诊/住院患者在医院的走向 - 病房到手术室的距离 - 工作场所布局的检查和调整 - 鼓励举例  分数：1□2□3□4□5□ | | | 患者来医院看病，需要经过哪些步骤？都在哪里完成这些步骤（如在哪儿挂号，在哪儿交费，如何办理住院）？病房与手术室距离如何？有患者专用电梯吗？常常会遇到布局方面的问题吗（例如需要耗材却无法及时获取，在病房经常找东西或取东西，护士站离医生办公室很远）会检查和调整布局吗？举个例子。 | | | | | | | |
|  |  |  | **1 分：**  医院整体布局和工作场所的布局/布置**不方便**患者就诊，如：病房与手术室不在同一楼层，医务人员需要耗材却无法及时获取。不为病人和员工考虑。没有开展减少布局问题的行动，或非常有限（例：政府要求） | | **3 分：**  医院整体布局经过全面考虑，以尽可能**最方便**患者的诊疗，但**不会定期**检查/调整工作场所的布局/布置；或者虽然在医院整体布局上**欠考虑**，但**会定期**检查/调整工作场所的布局/布置。尽可能的在优化患者流向；患者流向可能没有被常规的检查，但是**有努力**的去改善。 | | | **5 分：**  医院整体布局为患者的就诊提供最大方便，有足够权限的多角度检查组会**定期**检查工作场所的布局/布置是否便利，一旦需要，会**立即**做出调整。 | | |
|  |  |  | **2分**：医院布局**没有考虑周到**，但是对医院运转**没有负面影响**。减少布局问题的行动有限。  **4分**：医院布局**已经做到优化患者流向**；工作场所的布局和组织会常规的在**涉及不同级别员工**的会议上进行讨论。 | | | | | | | |
| 二、流程管理的理由  - 是否对流程进行改善 - 被动？迫于压力？主动？ - 改善的原因：财务压力？患者安全？ - 如何向员工传达这些理由和动机  分数：1□2□3□4□5□ | | | （如果受访者已经提到改善，直接问问题2）你们医院对患者就诊流程会进行改善吗？举个近期的例子你们多长时间会系统地讨论和检查一次就诊流程？有什么特别的因素让你们想要去改善就诊流程吗？（是政府部门要求，医院有财务压力，还是其他因素？）通常由谁来推动？员工知不知道改善的理由？参与程度如何？举个由员工推动的改善的例子。 | | | | | | | |
|  |  |  | **1 分：**  就诊流程的改善是**上级要求**的，或是由于**其他部门/科室**做出类似的改变，其**理由未与员工交流**，**员工不愿改变，什么都不做**。 | | **3 分：**  就诊流程的改善是迫于**财务压力，艰苦**和减少开支需要，或是作为一项（短期）措施，以达到政府和/或**外部目标（包括质量和满意度）。员工只有一点参与。** | | | **5 分：**  就诊流程的改善是为了提高医院**包括财务和临床的整体绩效**，这些变化的理由**连贯地传达**给员工，涉及到的**所有员工都同意，并积极参与改变。** | | |
|  |  |  | **2分**：做出了改善但是没有明显的效果。通常是**上级强加**的并且没有清晰的交流。  **4 分**：为了提高包括**财务和临床整体绩效**，**不同级别的员工**都贡献自己的力量以达到更好的效果。 | | | | | | | |
| 三、标准化诊疗  - 如何规范主要诊疗过程 - 应用情况和掌握程度 - 使用的工具和资源 - 如何监管应用情况  分数：1□2□3□4□5□ | | | 诊疗过程是怎么规范的？（例如不同的医生做同一个手术的过程是一样的吗？）举个具体的例子（骨外伤护理、支架手术）医务人员对于应该执行哪些具体的操作，掌握程度如何？怎么监督医生是按照已经制定的诊疗方案看病的呢？ | | | | | | | |
|  |  |  | **1 分：**  **没有**标准化的诊疗方案（如对于同一治疗，不同医务人员有不同的方式）；或者有一些指南但是没有标准化，也没有监督。 | | **3 分：**  制定了标准化的诊疗方案，但**没有被普遍使用**，且**监管手段单一。**（如只发布在网上或者手写版） | | | **5 分：**  全体员工都知道并使用标准化的诊疗方案，并进行**常规（每日）**监管/监督。有一个有效的监督系统并且会有报告。 | | |
|  |  |  | **2 分**：**制定了**标准化的诊疗方案，但**没有很好的沟通和使用**，可能有一点监督。  **4 分**：全体员工都知道并使用标准化的诊疗方案，并通过多种手段进行**常规**监管/监督，有一个监督系统能够发现问题。 | | | | | | | |
| 四、持续改进  - 发现和解决问题的**机制** - 员工在发现和解决问题过程中的**态度**和**参与程度**  分数：1□2□3□4□5□ | | | 一般你们是如何发现医院运营中的问题？如何解决这些问题？(用药差错、医务人员用很多时间填表格写报告等)。举个近期的例子发现和解决问题的动力是什么？医院内部通常哪些人参与解决问题的过程？ | | | | | | | |
|  |  |  | **1 分：**  **仅当问题发生时**，才会做出改进，或只**在一个类别员工**的内部解决（比如只涉及医生）。没有建立一个发现和解决问题的机制，一个问题解决了没有下一步措施，**没有员工的建议**。 | | **3 分：**  通过召开涉及**所有类别员工**的**不定期会议**发现和解决问题，讨论改进措施，要有**员工的反馈（正式或非正式）**。存在解决问题的程序，聚焦于找到解决方案，而非防止未来的问题。 | | | **5 分：**  在整个患者就诊过程中，通过**制度化**的方式发现/提出问题是员工的职责，解决问题需要**所有相关员工**参与；提出和解决问题是**日常工作的一部分。**持续改进是医院文化的一部分。 | | |
|  |  |  | **2 分**：问题发生的时候有一定的程序：**没有系统的正式的机制**，可能针对一些问题有指南可以参照；**关注改正而非改进**；在特别问题上**有一些员工建议**。  **4 分**：员工参与程度很高，有**制度化、系统化的**发现／提出问题的机制。持续改进程序聚焦于**防止问题发生**，而不仅是解决问题。医院鼓励员工去提出建议（有或没有奖励都行） | | | | | | | |
| 五、优化人力资源配置  - 如何安排员工工作 - 如何知道员工是否在岗位上发挥其优势 - 如何合理调配员工：如门诊高峰时段，有些科室比其他科室忙时 - 谁来调配，反应性的还是主动的  分数：1□2□3□4□5□ | | 如何知道不同的员工最适合做哪些工作？（职位描述、员工简历、人员能力列表）医院有些地方比其他地方忙的时候怎么办？采取什么措施来协助人员流动，例如有主要负责人或员工协调中心？ | | | | | | | |  |
|  |  | **1 分：**  员工通常**不能胜任**所做工作，或**大材小用**。即使有些员工很闲时，部门/小组间也**没有员工流动。**医院对紧急情况没有准备，不掌握员工的数量或技能，从来没有重新安排员工或者低效安排员工。 | | | **3 分：**  管理人员试图，但并**不努力确保**让员工发挥其长。员工在部门/小组间**有流动**，但经常**缺乏协调。**资深员工试图让员工做适合他的工作，会登记员工技能，但是没有一个明确的人来负责人员配置。 | | | **5 分：**  管理人员意识到有效部署人力资源是管理的核心，并**切实努力**优化人力资源。依据登记的技能表**常规协调**空闲的员工流动到忙碌的部门/小组。资深员工总是让员工做适合他的工作。员工流动是医院常规，并且是通过登记的技能表进行有组织有结构的调配。 | |  |
|  |  | **2 分**：管理者能够跨科室重新安排一些员工，但是不协调。**没有登记**员工技能，所以员工流动只是基于管理者安排。基本上**没有系统配置**。  **4 分**：资深员工总是让员工做适合他的工作。人员是**通过登记的技能表**进行**有组织有结构的调配**。有**专人**负责人员调配。 | | | | | | | |  |
| 六、绩效追踪  - 医院追踪了哪些绩效指标？哪些比较重要？ - 哪个部门负责追踪？ - 是否常态、是否有反馈？ - 是否公开？  分数：1□2□3□4□5□ | | 你们医院记录哪些工作指标？哪些指标比较重要？多长时间统计一次？有专门负责这项工作的部门或人员吗？哪些员工可以获得这些信息？在病房或手术室，能够直观的看到你们达到工作指标的情况吗？多长时间更新一次？ | | | | | | | |  |
|  |  | **1 分：**  绩效指标无法体现医院整体目标，**无绩效追踪或者不定期追踪**。**不与员工分享**。医院不追踪绩效，或者只关心基本的指标比如患者量。高级管理层才能看见指标。 | | **3 分：**  追踪**重要绩效指标**，追踪是**定期、正式地**进行，由**高层人员**监督、参与。**可视化的贴在某个地方**，员工可以看到。可以公开或者不公开，更新不及时或不有效公开）。 | | | **5 分：**  **持续、定期追踪**重要绩效指标，**直观手段，正式或非正式**的形式向**全体员工**传达。**每天都更新**。所有指标都正式且持续追踪；记录会被自动在电脑系统上更新，所有的员工可以查看。在很多的地方都能看到各自的表现。 | | |  |
|  |  | **2 分**：不定期追踪重要绩效指标，**仅在员工邮件中分享；或**正式追踪有限的绩效指标，中高层可见。  **4 分**：每日追踪**大部分指标**。管理层都能看见。主要指标**更新频繁（周，月）**。**员工主动**去查看他们的表现。 | | | | | | | |  |

| 七、绩效考核  - 绩效考核方法是什么，此处对于临床科室人员可能并不清楚，可大概描述考核过程 - 考核完毕是否有后续的跟进措施 - 结果是否通报给全体员工  分数：1□2□3□4□5□ | 你们如何考核达成这些工作指标的情况？多久开展一次考核工作？哪些人可以知道考核结果？工作考核后，有什么样的后续跟进措施？（不关注对人的惩罚，而是如果发现问题进行改进） | | |
| --- | --- | --- | --- |
|  | **1 分：**  绩效考核**太粗略**，只关注**达成或未达成**绩效目标；或**不定期地**进行绩效考核，以非正式或临时方式。 | **3 分：**  **定期**开展绩效考核，结果汇报给**高层管理人员**，**无明确的后续跟进**措施。即使有也**不够正式**。 | **5 分：**  **持续、定期**考核，考核所有方面都有**后续跟进措施**，结果会**主动并有效的传达给全体员工**（可视化显示，会议等） |
|  | **2 分：定期**进行绩效考核，但是讨论的项目很有限，**周期太长**（超过一个季度），没有后续计划。若是员工会议，则不向高级管理层交流结果；若是管理层会议，则不向员工交流结果。  **4 分**：有正式的、**结构化的后续计划**，包括时间、哪些人、做什么。还要有**监督计划的进展**。 | | |
| 八、绩效沟通  - 绩效讨论会是否定期举行 - 绩效讨论会是否提出适当的绩效数据，并且根据数据找出问题的根本原因和解决措施 - 所有与会人员是否明确了解会议议程、目标、后续计划 - 会议是否提出建设性反馈意见并有指导作用  分数：1□2□3□4□5□ | 您参与过刚才提到的工作考核会议吗？这些会议是如何开展的？哪些人参与？会上提供哪些信息？ 您觉得会上提供了足够的信息进行绩效考核吗？您认为这些会议有多大用处？会上有哪些反馈？对于发现的问题，你们如何找到根本原因？ | | |
|  | **1 分：**  绩效讨论中**未涉及**到实质性信息或**信息质量过低**。**没有**明确的**议程、目标**，也**没有**提出**后续**计划。  会议没有结构，议程未知；没有明确的会议目的。很多或者很少的数据。 | **3 分：**  绩效讨论会议建立在**适当的绩效数据**上，所有与会人员都**明确**会议**目标和议程**，但讨论**没有找出**导致问题的**根本原因**，并且**没有**明确提出**后续计划**。会议议程和结构都很明确。合适的相关数据。没有根因分析。 | **5 分：**  有**定期**的绩效讨论**会议**，针对导致问题的**根本原因**和**解决措施**进行讨论。所有与会人员都**明确**了解会议的议程、目标和后续计划。会议可以提供建设性**反馈意见**，同时具有**指导作用**。4分的基础上，会议不仅关注好的方面也关注坏的方面（为了**保持好的**，并**避免坏的**再发生）。 |
|  | **2 分：**有一定的会议议程／结构。一些KPI会被讨论，很多或者很少的数据。  **4 分**：有合适的绩效数据在会上沟通，与会人员都**明确会议议程**，大多数能使用根因分析的方法找到**根本原因**，能明确提出后续改进计划。 | | |

| 九、结果管理  - 处理是否及时 - 是否正式的处理措施 - 是否有专门人员负责  分数：1□2□3□4□5□ | | 假设在刚才提到的工作讨论会中，你们科室同意一项后续跟进措施，如何知道后续计划的进展？如果这个措施后来没有被执行，会如何处理？从发现问题到解决问题间隔多长时间？举个最近的例子如果某科室反复未能达成工作指标，你们是如何处理的呢？ | | | | | | | |
| --- | --- | --- | --- | --- | --- | --- | --- | --- | --- |
|  |  | **1 分：**  未能完成后续计划，却**不**用承担任何后果。  没有采取行动。在运营上没有任何改变。 | | | | **3 分：**  有**监控过程了解后续计划的进展**。未完成后续计划，**有**应对改进**措施**，**没有及时**采取应对改进措施。  能通过**常规会议**发现失败（周，月例会）或者在截止日之前的时间点发现。可以**调整计划**以期能在规定时间内**达成目标**。 | | | **5 分：**  未完成后续计划，会**立刻**采取**规范的处理措施**，并有**专门行动小组**负责监督、完成处理措施。**重新培训**调**岗**。 4分基础上，**多种工具**会自动检查和报告给负责的管理者。在会议（正式或非正式）上会查看根本原因，以及采取措施来**防范于未然**。 |
|  |  | **2 分：**管理层**对后续计划的进展不知情**，不知道需要做什么。只有在截止日时才会发现没有达成目标，但还是做出**一些努力**来尽可能的进行改变。  **4 分：管理者会在一定时间线（截止日之前）上追踪**后续计划的**进展**。会**移动资源（例如人力）来解决问题**。 | | | | | | | |
| 十、目标平衡  - 医院的目标 - 设定目标的动机：上级要求？医院自身需要？ - 员工对目标的了解程度  分数：1□2□3□4□5□ | | 医院有哪些方面的具体目标？这其中哪方面的目标强调得比较多？具体说说那些不是根据外部要求（如政府和某些规定）设定的目标？这些目标之间的关系是什么？（员工是否理解？） | | | | | | | |
|  |  | **1 分：**  医院只关注政府设定的总体目标。只有财务和运营目标。 | | | | **3 分：**  总体目标**平衡了质量、等待时间、效率、和财务等具体目标**；总体目标只是评估高层管理人员，没有扩展到所有员工。并非优先考虑非财务目标，没有很好地与所有员工交流。各项目标之间的相互依从性没有被很好理解。 | | | **5 分：**  总体目标由若干子目标构成，并且保持平衡，涵盖了若干维度，例如运营、财务、医护满意度、患者满意度。所有员工的评估均包含目标设定。各级员工（临床医师、护士、管理者）都能很好理解上述目标间的相互作用机制。财务和非财务目标之间很好的平衡。**非财务目标**可能会更重要，因为**会影响到医院长期发展**。 |
|  |  | **2 分：有一些非财务目标**，但都是**政府规定**的（没得选）。  **4 分：**具体的**非财务目标**是管理层奖励的重要部分，因为这些目标**是优先考虑的**。员工能够**很好理解**各项目标的关系。 | | | | | | | |
| 十一、目标的相互联系  - 医院整体目标如何落实到个人、科室 - 是否了解个人目标、科室与医院目标的关系 - 鼓励举例  分数：1□2□3□4□5□ | | 就您提到的医院所有这些目标，制定的动机是什么？你们医院整体目标是如何传达并分解到各层级员工的？ （是否分解到不同层级员工？是否不同层级员工都有更小的目标？）科室目标是如何与医院整体目标联系？可否举个例子？ | | | | | | | |
|  |  | **1 分：**  医院总体目标**未传达**或未分解。 | | **3 分：**  医院总体目标传达给所有人，并分解到**管理层/科室/部分类别员工（只是护士）**。 | | | **5 分：**  目标分解得越来越细，最终**对每个人都有预期**。医院目标清晰地传达和分解到所有级别。总体目标是基于**利益相关者利益**设定的。 | | |
|  |  | **2 分：**医院总体目标**只传达到部分管理层**，没有再向下传达。  **4 分：**医院总体目标清晰地**传达和分解到所有级别**。管理者提到**股东利益**很重要。 | | | | | | | |
| 十二、目标时限  - 医院的长期目标、短期目标 - 科室的长期目标、短期目标 - 长期目标和短期目标的受重视程度 - 短期目标与长期目标的关系  分数：1□2□3□4□5□ | | 你们医院目标有哪些时限（几个月？一年？几年？）哪种目标（短期、长期目标）最受重视？短期目标和长期目标之间的联系是怎样的？会不会实现了所有的短期目标，却没有达到长期目标？ | | | | | | | |
|  |  | **1 分：**  只关注短期目标的实现。只有不超过6个月的短期目标 | | **3 分：**  医院的各个部门\科室都制定了短期和长期目标。短期和长期目标是独立制定的，两者之间没有必然的联系。1没有清晰的大于一年的计划，但是这一年的目标间紧密联系。2有超过一年的目标，但是长期目标对他们而言毫无意义。 | | | **5 分：**  长期目标被分解成一系列具体的短期目标，短期目标成为了实现长期目标的“阶梯”。 清晰的多年度战略计划和年度目标。短期，中期和长期目标很好的联系在一起（阶梯）。所有不能预见的改变会让他们评估短期目标，以保证目标间相关联。长期目标经常达成。 | | |
|  |  | **2 分：**有年和月目标。**关注月目标**。  **4 分：**清晰的多年度战略计划和年度目标。所有目标都是**相关的**，但是可能没有很好的联系起来。 | | | | | | | |
| 十三、目标难度  - 科室目标的**难易程度** - 目标的**制定标准** - 科室、部门、员工间目标**难易程度均衡**  分数：1□2□3□4□5□ | | 这些目标给你带来的压力如何？通常，能达到目标的机率有多高？这些目标是依据什么标准制定的？（有没有参考医务人员的意见？）您觉得所有科室、部门或不同职能员工的目标难易程度是一样的吗？（是不是有些员工的目标比较容易达到？） | | | | | | | |
|  |  | **1 分：**  目标太容易或太难，部分原因是由于没有考虑医务人员的意见，例如设定目标仅仅参考以往业绩数据等。太难（－60%）或太简单（＋90%） | | **3 分：**  根据外界标准制定难度适中的目标，几乎没有参考医务人员的意见。部分科室/员工不受目标约束（Sacred Cow）。管理者觉得压力较大，完成度介于80%－90%。完成目标上**不同科室有不同程度的困难**。 | | | **5 分：**  目标的设定综合考虑了各部门情况，并在咨询高层员工意见的基础上形成，例如根据内部和外部标准进行适度调整，目标难度在各部门间均衡。4分基础上，**定期检查目标以考虑经济状况和面临的挑战**。 | | |
|  |  | **2 分：**目标太难或者太容易，**有一定的制定依据**，比如根据以往业绩。管理者觉得压力很大。完成度介于60％-70％。一些科室有相比其他科室更容易达成的目标  **4 分：**目标的设定考虑到了各部门之间的**差异**，但是在收集员工的意见上比较的被动。管理者觉得压力很大，完成度介于80%－90%。完成目标上**所有科室有相同程度的困难度**。 | | | | | | | |
| 十四、目标清晰度和可比性  - 员工是否清楚个人目标 - 目标清晰度？复杂程度？ - 部门绩效公开与否？鼓励竞争？  分数：1□2□3□4□5□ | | 如果直接问员工你们医院的每一个具体目标，他们会如何回答？（是否知道？是否经过沟通？）有人抱怨目标太复杂、不太容易理解吗？（举例子？）人们如何知道他人的工作表现？（会采用什么方式公布吗？有排名吗？） | | | | | | | |
|  |  | **1 分：**  目标太复杂且不易理解，不被公开 | | **3 分：**  目标经过沟通与清晰定义；工作表现（组或个人）公开，但是不鼓励比较。 | | | **5 分：**  目标经过沟通与清晰定义；工作表现（组或个人）公开，通过**公开排名鼓励个人层面上的比较**。 | | |
|  |  | **2 分：**目标**很清晰**。以组为单位，而不是个人。不公开表现。  **4 分：**目标经过沟通与清晰定义；工作表现（组或个人）公开，鼓励以组为单位进行比较或者非正式的个人层面上的比较。 | | | | | | | |
|  | |  |  |  |  |  |  |  |  |
| 十五、奖励优秀员工  - 员工收入和员工工作水平的联系,即有无与工作表现相关的奖励评估体系 - 有无非奖金形式的奖励  分数：1□2□3□4□5□ | | 员工评价/审核体系是怎样运作的？能告诉我最近一轮的情况吗？如何根据上述审核结果支付员工？奖金体系是如何运作的？表现最优秀的员工有非奖金形式的奖励吗？你们的奖励体系与其他同类医院相比怎么样？ | | | | | | | |
|  |  | **1 分：**  员工**获得同等的奖励**，与个人工作表现无关。管理者和员工的奖励都一样。 | | **3 分：**  存在和个人工作表现相关的**非奖金和奖金**奖励评估体系。但奖励总是**很容易或者很难达到**。管理者和员工都是基于工作表现，或者，管理者有**非常好的奖励和奖金体系**，而员工都是一样。 | | | **5 分：**  存在与工作表现相关的奖励评估体系，包括个人奖金与非奖金的奖励。有**合理的奖励机制**，对个人工作表现有明确的定义和监管。管理者和员工都是基于工作表现。有财务和非财务奖励。有非常好的评价系统（更多的常规评审，并且强力和平衡的测评） | | |
|  |  | **2 分：**一部分人相同，**一部分人（如管理者或普通员工）根据表现，**或者组内相同**。**  **4 分：**管理者和员工都是基于工作表现，有财务和**非财务奖励**。或者管理者和员工都是基于工作表现，**非常好的评价系统**（更多的检查，并且强力和平衡的测评）进行财务奖励。 | | | | | | | |
| 十六、调离表现欠佳的员工  - 如何处理表现不佳的员工，是否有口头警告/书面警告/终止合同 - 从发现表现不佳的员工到对其进行处理的时间间隔是否超过一年 - 是否有表现欠佳的员工能够逃避调离处理 - 既包括工作能力差的员工，也包括工作态度差的员工  分数：1□2□3□4□5□ | | 如果遇到医生或者护士无法胜任其工作，你们会如何处理？能否举一个近期发生的例子？员工如果表现欠佳，你们多久会采取措施？（比如调离岗位或是辞退？）解雇一名护士/医生的难度有多大？有没有一些表现欠佳的员工过得非常舒服，总能避免被“处理”？ | | | | | | | |
|  |  | **1 分：**  表现欠佳的员工**几乎不**会被**调离**职位 | | **3 分**：  表现欠佳的员工继续在该职位工作**超过一年**后医院才会采取措施。有**正式的程序**如警告---改进计划---再警告等。会通过评价系统发现表现差的员工，会在几个月之后解雇或调岗。个别人会使用手段留下。 | | | **5 分：**  一旦发现表现欠佳的员工，会**立即**被医院辞退，或调离科室，或者被安排至一个不重要的职位，并在**三个月内**就会采取措施。会通过常规评价系统发现表现差的员工，会立即进行正式的提高帮助计划，适用于所有员工。 | | |
|  |  | **2 分：**表现差的员工会在几年之后**解雇或调岗**。没有主动发现这些人的系统。个别人会使用一些手段留下。  **4 分：**会通过常规评价系统**发现表现差的员工**，会立即进行帮助（培训等），会待岗6个月左右。适用于所有员工。 | | | | | | | |
| 十七、提拔优秀员工  - 提拔员工是以工龄还是以工作表现为依据 - 如何发现和培养优秀员工 - 提拔的培养手段可包括训练课程、支付再教育费  分数：1□2□3□4□5□ | | 你们医院的（行政）职位晋升体系是怎样的？职位晋升的频率如何？你们如何发现和培养非常优秀的员工？为优秀员工提供什么样的职业发展机会？在医院和科室内你们如何做晋升的决定？表现好的员工更可能得到较快的提拔，还是根据年资？ | | | | | | | |
|  |  | **1 分：**  **只基于工龄**提拔。没有晋升系统（医院很多年没有人被提拔了）；或者基于工龄。 | | **3 分：**  晋升系统根据**工作表现**：医院可能有着**外部局限**性（工会），但是可以打擦边球（给予更多的责任或专业晋升）；或者医院可能有**内部局限**性（很少的职位空缺），但是可以打擦边球（额外的培训）。 | | | **5 分：**  医院积极地发现、**培养**和提拔表现优秀的员工。晋升基于工作表现。医院**主动发现**，发展和提升表现好的员工。个人有常规评估、明确的指标和个性化的**职业发展规划**（定期进行修改）。 | | |
|  |  | **2 分：晋升基于工作表现和工龄**，或晋升系统基于工作表现，但是**很多年**没有一个人被提拔了（3年以上）。  **4 分：**医院通过**系统的评价发现**工作表现好的晋升。向重点培养员工提供额外的**培训**。真正的晋升机会向所有员工开放。 | | | | | | | |
| 十八、人才管理  - 管理人员如何确保合适和充足的人才使用，比如有详细的工作描述，校园招聘，员工推荐 - 管理人员是否将吸引和培养人才的理念传达给员工 - 管理人员是否对构建人才库问责并获得相应奖励，人才流动率、人才保留率是否是评价管理人员的指标之一  分数：1□2□3□4□5□ | | 医院如何确保人岗匹配，并且员工充足？管理人员如何让大家知道吸引人才和让人才发挥最大作用是医院工作的重点？管理人员因为引进或留住优秀人才而获得任何奖励了吗？ | | | | | | | |
|  |  | **1 分：**  高级管理者**不**将吸引、留住和培养优秀人才作为医院工作的重点 | | **3 分：**  高级管理者相信拥有优秀人才是确保良好绩效的关键，并将这种理念**传达**给员工。但是对管理者**没有正式的问责机制**。 | | | **5 分：**  对管理人员的评估基于主动构建人才库的力度，并有**明确的问责制度。**吸引和发展人才通过目标和奖励（财务和非财务）来正式化。吸引和发展**所有层级的人才**是高级管理者奖励系统的一部分。 | | |
|  |  | **2 分：**高级管理者理解这是**重点**，但是**没有程序或者正式交流过**。  **4 分：吸引和发展人才通过目标和／或奖励来正式化**，吸引和发展**优秀管理者**是高级管理者奖励系统的一部分。 | | | | | | | |
| 十九、人才保留  - 医院如何处理打算离职的人才 - 有没有尝试一切可能方式留住人才 - 有没有涵盖所有类别的员工/人才 - 这里指的是最顶尖的人才  分数：1□2□3□4□5□ | | 假如一个优秀的医生、护士或者管理人员打算辞职，医院会怎么办？能否举个医院试图挽留杰出员工的例子？ | | | | | | | |
|  |  | **1 分：**  **几乎没**采取措施来留住优秀人才。医院不做任何事来留住优秀人才。 | | **3 分：**  **努力**留住优秀人才。医院会谈判，提供稍微好一点的条件，但是很局限。 | | | **5 分：**  尝试**一切可能的方式**留住所有类别员工中的优秀人才 | | |
|  |  | **2 分：**医院**试着去**理解优秀人才为什么想离开（为什么想走），没有进一步行动。  **4 分：**医院提供了一系列的**福利包**来留住优秀人才**。不仅是和其他医院提供相同，而且要更好的待遇来留人。** | | | | | | | |
| 二十、人才吸引  - 医院提供的员工价值主张包涵哪些内容 - 与同类型医院（竞争对手）比较，有何独特性 - 顶尖人才为什么愿意在此工作  分数：1□2□3□4□5□ | | 与其它医院比较，你们医院为吸引人才提供了哪些奖励、福利、或发展机会？假如我是一个很优秀的护士或医生，您如何说服我加入你们医院？与其他医院比，您认为你们医院的员工可能不喜欢在你们医院工作的理由是什么？您给自己的工作幸福感打个分？1分最差，5分最好。 | | | | | | | |
|  |  | **1分：**  **竞争对手**为优秀人才提供**更有力**的条件使人才加入，医院**没有**提供自己的**员工价值主张。**没有激励系统来吸引优秀人才。 | | **3分：**  医院的员工价值主张**与其他医院差不多。**激励系统比较起来有优势并且／或者好到可以吸引一些优秀人才。 | | | **5分：**  为了吸引人才，医院提供**独特**的员工价值主张以鼓励**顶尖**人才加入。 | | |
|  |  | **2 分：**有条件做到与竞争对手提供**差不多**的待遇，但是没有自己的价值主张。有一定的激励系统，但是没有竞争力（不是最好的工作场所）。  **4 分：**为了吸引人才，做的**比一般的医院更好**，有优厚的待遇吸引人才**。** | | | | | | | |

注：每个问题得分为1到5分，其中1分为最低分，5分为最高分。可以用某一维度内所有管理实践的平均得分来反映该维度得分情况。管理得分总分为20个管理实践得分的平均分。
